# Psychotherapeutic and work-oriented interventions: employment outcomes among young adults with work disability due to a mental disorder

**DOI:** 10.1186/s13033-016-0101-7

**Published:** 2016-10-12

**Authors:** Pauliina Mattila-Holappa, Matti Joensuu, Kirsi Ahola, Aki Koskinen, Katinka Tuisku, Jenni Ervasti, Marianna Virtanen

**Affiliations:** 1 Finnish Institute of Occupational Health, Töölöntullinkatu 8, 00250 Helsinki, Finland; 2 Outpatient Clinic for Assessment of Ability to Work, Department of Psychiatry, University of Helsinki and Helsinki University Central Hospital, Helsinki, Finland

**Keywords:** Mental disorder, Occupational, Prospective, Psychotherapy, Work, Vocational, Intervention, Young adults

## Abstract

**Background:**

We examined the extent to which psychotherapeutic and work-oriented interventions were included in a medical treatment and rehabilitation plan and whether they predicted future employment among young adults with work disability due to a mental disorder.

**Methods:**

Data were obtained from the treatment and rehabilitation plans of 1163 young adults aged 18‒34 years, who in 2008 were granted fixed-term work disability compensation due to a mental disorder and were followed for 5 years.

**Results:**

Forty-six percent had no proposal for psychotherapy or a work-oriented intervention in their treatment and rehabilitation plan, 22 % had a plan for only a psychotherapeutic intervention, 23 % had a plan for only a work-oriented intervention, and 10 % had both types of interventions planned. Having a planned psychotherapeutic intervention (HR = 1.35, 95 % CI 1.07–1.69) and of the work-oriented interventions, planned rehabilitative courses and training (HR = 1.34, 95 % CI 1.03–1.70) predicted quicker entry into competitive employment. Having a plan for both a psychotherapeutic and work-oriented intervention was associated with being employed at the end of the follow-up (OR = 1.77, 95 % CI 1.07–2.95).

**Conclusions:**

Young adults with a long-term psychiatric work disability episode rarely have a recorded plan for rehabilitation in their treatment and rehabilitation plan although psychotherapeutic interventions and a combination of a psychotherapeutic and work-oriented intervention might help them gain employment.

## Background

Mental disorders comprise the majority of the causes of work disability among young adults [1–4]. Since disability at an early age incurs high costs for society [1, 5] and may lead to exclusion from society at an early stage of life, it is crucial that means to facilitate the integration of young adults into the labour market are developed.

In Finland, in 2014, 76 % of new disability pensions granted to young adults (18‒34 years) were due to mental disorders [6]. Disability pension may be granted after 300 days of sickness absence. Pensions for young adults are usually granted as fixed-term (e.g. for a year), since these young adults are expected to return to employment. During this fixed-term work disability period, rehabilitation should be systematic and intensive in order to foster returning to work or studies.

Previous studies have shown that the risk factors that predict a low probability of returning to work after a period of disability due to poor mental health include work-related factors (e.g. temporary employment), social status and demographics (e.g. older age, unemployment), and health risks (e.g. drug dependence), and clinical factors (e.g. diagnosis and severity of disorder) [7–11].

In addition to medical treatment, psychotherapy may promote recovery from mental disorder and also help in the return to employment. After long-term absence from employment, recovery from illness alone does not necessarily ensure entry or return to employment, because it may be difficult to find suitable work, work should be modified to correspond work ability, and social support during integration to employment is needed. Work-oriented interventions which are planned to assist integration to employment, may include for example group or individual training to support employment, work trials in which the client works as an extra worker in competitive work and is compensated by state, and job modification (e.g. modifying working hours or excluding tasks which require undisturbed concentration).

Individual-focused psychotherapeutic interventions have shown to be effective in treating a wide range of mental disorders among young adults [12, 13] and seem to enable competitive employment of psychiatric patients without work disability at baseline [14]. In Finnish treatment guidelines [15] psychotherapeutic interventions are recommended in the treatment of mild to moderate or severe depression. Cognitive or cognitive-behavioral therapy is seen as possible treatment also in bipolar and psychotic disorders. However, it is not known if these psychotherapeutic interventions help young adults gain competitive employment, and if the effect is associated with diagnosis.

Little evidence currently exists on the efficacy of work-oriented interventions, which, often without close contact to workplaces, focus on preparing participants for employment [16]. The individual placement and support model (IPS) however, which unfortunately is not largely applied in Finland, has been effective in helping people with severe mental illness gain competitive employment [17–19], also young adults [20]. In Finland, occupational pension institute-funded ‘vocational rehabilitation’, which is also executed with close contact to work places, has shown to be successful in terms of return to work [21]. However, most young adults with long-term work disability due to mental disorders were poorly attached to employment before the work disability period [22]. The problem also is that they usually do not fulfil the criteria (a total of 34,508 € of earnings during the past 5 years) for this vocational rehabilitation. Overall, the rehabilitation of young adults is organized by various service providers, but the effectiveness of those interventions is not known.

Few previous studies on interventions and employment outcomes have focused particularly on young people. For young adults, the period on fixed-term work disability allowance may be a critical period in view of possibilities of returning to employment as opposed to permanent pension. Effective rehabilitation should thus be organized during this period. However, there is little information on interventions that could promote employment among young adults with mental disorders or if the diagnosis is associated with intervention results. The aim of this study is to examine whether psychotherapeutic and work-oriented interventions targeted towards young adults on fixed-term work disability pension due to a mental disorder are associated with employment outcomes over 5 years.

## Methods

The study is part of the Young Minds at Work Study [23] which aims to determine the factors associated with work disability due to a mental disorder and re-entry into the labour market or education of young adults in Finland. The study was approved by the Ministry of Social Affairs and Health, and the Ethical Committee of the Hospital District of Helsinki and Uusimaa. As our study was based on register data, informed consent from the subjects was not obtained. The sample comprised all people aged 18‒34 who received fixed-term work disability pension due to a mental disorder from occupational pension institutes in 2008 in Finland (N = 1163). This allowance is granted after 300 days of disability, usually for 1 year at a time. During the allowance period, rehabilitation should be planned and carried out. Applications for disability pension must be accompanied by a medical certificate which specifies treatment and a rehabilitation plan. All of the young adults studied were receiving treatment in a psychiatric clinic, health centre, or occupational health care, and 98 % of them had been prescribed psychotropic medication [23].

The inclusion criteria was work disability pension granted due to mental disorders according to the International Classification of Diseases, tenth revision ICD-10. We included ICD 10-codes F10–F69 and F80–F99 as the primary cause of work disability. Cases with F00–F09 (organic mental disorders) and F70–F79 (mental retardation) diagnoses were excluded.

Three researchers collected the data from the pension institutes between September 2012 and June 2013. They used a structured Excel sheet, which was later transformed into a quantitative dataset. The researchers coded 40 cases as duplicates to assess inter-rater reliability. The mean agreement between researchers was 92 %.

The data on psychotherapeutic and work-oriented interventions were collected from patients’ disability pension applications and the attached medical certificates: *Planned psychotherapeutic intervention* (yes/no), to be carried out by a trained psychotherapist according to the medical treatment and rehabilitation plan, and *Planned work*-*oriented intervention* (yes/no).

This included the following interventions: (1) Assessment of working capacity and evaluations of rehabilitation needs. These evaluations are usually made in hospital or in rehabilitation institute, and they are made in co-operation of several professionals: physician, occupational therapist, psychologist and social worker. (2) Rehabilitative courses and training. These included group and individual training to enhance working capacity, knowledge of working life and contacts with potential employers. The training may be organized by occupational pension institutes, Social Security Institution or employment offices. (3) On-the-job rehabilitation. This included work trials in which the client works as an extra worker in workplace with a salary compensated by insurance company or employment administration. (4) Social rehabilitation. This included rehabilitative work and club house activities, which do not necessarily aim to competitive employment, but serve the aims of rehabilitation, meaningful activity, and social interaction.

The outcome, employment status data, were derived from the National register of the Finnish Centre for Pensions: (1) *Entry into employment; time to first day of employment*, i.e., number of days from the first day of work disability pension to the first day in employment or to the end of the follow-up period (31 December, 2013); (2) *Employment status at the end of follow*-*up:* employed versus not employed at the end of the follow-up, 31 Dec. 2013.

Covariates included *Sex and age* (age classified as 18‒24, 25‒29, 30‒34); *basic education* (comprehensive school, upper secondary school) and *vocational education* (no vocational education, vocational course or apprenticeship, vocational school, university of applied sciences, university level/Master’s degree); and *primary diagnosis* according to the ICD-10 classification, further categorized as psychotic (F20–F29), depressive (F32–F34), bipolar (F30–F31), or other mental disorder (F10–F19 and F40–99). The most common diagnoses in the ‘other mental disorder’ category were *neurotic, stress*-*related and somatoform disorders* (F40–48, N = 76/137); *psychiatric hospital admission:* at least one/none, based on medical records; and *attachment to employment before work disability*: employment records from the Finnish Centre for Pensions (number of days of competitive employment during the 3 years preceding work disability pension). Those with 730 or more days (2 years) of employment during the 3 years preceding the date when work disability compensation started were considered attached to employment.

### Statistical analyses

We first calculated the mean, standard deviation, and percentage at the end of follow-up, and at some time during follow-up, for employment, full-time disability pension, part-time disability pension, and receipt of other benefit (e.g. unemployment benefit).

The associations between entry into employment and the independent variables were: (1) psychotherapeutic intervention vs. neither intervention (=no psychotherapeutic, no work-oriented), (2) work-oriented intervention vs. neither intervention, or (3) both psychotherapeutic and work-oriented intervention vs. neither intervention. These were assessed using Cox proportional-hazards models. For each participant, the follow-up person-days were calculated as those from the first day of work disability pension to the first day in employment or to the end of the follow-up period (31 December, 2013), whichever came first. The time-dependent interaction terms between each predictor and logarithm of the follow-up period were non-significant, confirming that the proportional hazards assumption was justified (all *p* values >0.70). The hazard ratios (HRs) and their 95 % confidence intervals (95 % CIs) for categorical independent variables provided risk estimates.

We conducted the analysis in three stages: Model 1 was adjusted for age and sex. Model 2 was adjusted for age, sex, type of diagnosis (psychosis, depression, bipolar, and other) and psychiatric hospital admission. Model 3 was adjusted for all the factors in Model 2, and additionally for attachment to employment before work disability pension. Following a similar procedure, the associations between the independent variables and employment status (employed vs. not) at the end of the follow-up on 31 December, 2013 were assessed using binary logistic regression models.

The analyses were repeated in groups determined by basic education, attachment to employment before work disability pension, and diagnosis category with both employment outcomes. Finally, we assessed the associations between the four types of work-oriented interventions: (1) assessment and evaluation, (2) rehabilitative courses and training, (3) on-the-job rehabilitation, and (4) social rehabilitation vs. no intervention, and employment outcomes, using Cox proportional-hazards models and logistic regression. The statistical analyses were performed using phreg and logistic procedures in SAS 9.4, SAS Institute, Cary, NC [24].

## Results

Table 1 shows the descriptive results for different indicators of employment during the 5-year follow up. At the end of the follow-up, 22.0 % were employed, whereas 45.0 % were on full disability pension. A total of 48 % had undergone a period of employment at some time during the follow-up. On average, the young adults were employed for 377 days during the 5-year follow-up, which is 27 % of the total follow-up time. The mean time to the first day of employment after the start of the fixed-term work disability pension was 710 days.

**Table 1:** Employment status of young adults during 5-year follow up period

Employment status	Mean (days)	Standard deviation (days)	Per cent at end of follow up	Per cent at some time during follow up
Full-time disability pension	1400.2	806.3	45.0	100.0
Part-time disability pension	43.5	263.8	1.9	3.5
Employment	377.0	593.4	22.0	48.0
Other benefit	334.0	532.4	16.9	43.3

In their treatment and rehabilitation plan, altogether 540 (46.4 %) the young adults had no plan for psychotherapy or work-oriented intervention, 250 (21.5 %) had a plan for a psychotherapeutic intervention (no work-oriented intervention), 263 (22.6 %) had a plan for only a work-oriented intervention, and 110 (9.5 %) had a plan for both types of interventions (data not shown in tables).

The associations of planned interventions with entering employment and with employment status at the end of the follow-up are presented in Table 2. The Cox proportional hazard models showed that after adjusting for age, sex, basic education, diagnosis, psychiatric hospital admission, and attachment to employment before work disability pension, planned psychotherapeutic interventions were associated with quicker entry into employment (HR = 1.34, 95 % CI 1.06–1.68) than that of those for whom neither of the interventions was planned. Work-oriented interventions as a whole were not associated with employment outcomes after full adjustments. However, the analyses of separate types of work-oriented interventions (full data not shown) showed that a plan for ‘rehabilitative courses and training’ was associated with quicker entry into employment sooner than no planned interventions (HR = 1.34, 95 % CI 1.03–1.75). On-the-job rehabilitation was associated with earlier entry into employment after adjusting for sex, age, basic education, diagnosis, and psychiatric hospital attendance (HR = 1.52, 95 % CI 1.14–2.02), but the association was not significant after adjusting for attachment to work before work disability. Those with a plan for both a psychotherapeutic and a work-oriented intervention were more often employed at the end of the follow-up (OR = 1.77, 95 % CI 1.07–2.95) than those for whom neither intervention was planned.

**Table 2:** Associations of intervention plans with entering employment during follow-up and being employed at end of follow-up

Planned intervention		Entering employment during follow-up							Being employed at end of follow-up						
		N (cases)	Model 1		Model 2		Model 3		n (cases)	Model 1		Model 2		Model 3	
			HR	95 % CI	HR	95 % CI	HR	95 % CI		OR	95 % CI	OR	95 % CI	OR	95 % CI
Psychotherapeutic intervention	Neither^a^	540 (223)	1.00		1.00		1.00		540 (95)	1.00		1.00		1.00	
	Only	250 (148)	1.55	(1.25–1.92)	1.35	(1.07–1.69)	1.34	(1.06–1.68)	250 (76)	1.87	(1.31–2.67)	1.35	(0.92–1.98)	1.32	(0.89–1.43)
Work-oriented intervention	Neither^a^	540 (223)	1.00		1.00		1.00		540 (95)	1.00		1.00		1.00	
	Only	263 (126)	1.25	(1.00–1.55)	1.24	(0.99–1.55)	1.17	(0.94–1.46)	263 (50)	1.12	(0.77–1.65)	1.06	(0.72–1.58)	1.02	(0.68–1.52)
Psychotherap. and work-oriented intervention	Neither^a^	540 (223)	1.00		1.00		1.00		540 (95)	1.00		1.00		1.00	
	Both	110 (61)	1.41	(1.07–1.89)	1.34	(1.00–1.81)	1.27	(0.94–1.72)	110 (35)	2.11	(1.33–3.35)	1.79	(1.10–2.91)	1.77	(1.07–2.95)

Model 1: adjusted for age and sex

Model 2: adjusted for age, sex, basic education, diagnosis, and hospital admission

Model 3: adjusted for age, sex, diagnosis, hospital admission, and attachment to work preceding pension

^a^Reference group: no psychotherapeutic, no work-oriented intervention

The associations between planned interventions and future employment are presented in Table 3, by basic education and by attachment to employment before work disability pension. The results show that among those with a high school education, having a plan for both interventions was associated with earlier entry into employment than having no planned interventions (HR = 1.66, 95 % CI 1.07–2.57). In addition, among those who were attached to employment before work disability, having a plan for both interventions was associated with being employed at the end of the follow-up (OR = 2.76, 95 % CI 1.26–6.02).

**Table 3:** Associations between intervention plans and employment outcomes by education and attachment to work

Education/attachment to work before disability	Planned intervention	Entering employment during follow-up							Being employed at end of follow-up						
		n (cases)	Model 1		Model 2		Model 3		n (cases)	Model 1		Model 2		Model 3	
			HR	95 % CI	HR	95 % CI	HR	95 % CI		OR	95 % CI	HR	95 % CI	HR	95 % CI
	Psycho-therapeutic intervention (PI) vs. neither														
Comprehensive school	Neither	339 (130)	1.00		1.00		1.00		339 (52)	1.00		1.00		1.00	
	PI	110 (55)	1.39	(1.01–1.92)	1.21	(0.86–1.70)	1.32	(0.94–1.84)	110 (28)	1.80	(1.05–3.07)	1.43	(0.82–2.49)	1.53	(0.87–2.68)
High school	Neither	187 (88)	1.00		1.00		1.00		187 (42)	1.00		1.00		1.00	
	PI	139 (92)	1.55	(1.15–2.08)	1.51	(1.10–2.08)	1.38	(1.00–1.90)	139 (47)	1.66	(1.01–2.74)	1.29	(0.75–2.22)	1.18	(0.68–2.05)
Attached to work	Neither	154 (103)	1.00		1.00				244 (75)	1.00		1.00			
	PI	99 (80)	1.38	(1.03–1.87)	1.32	(0.96–1.81)			138 (64)	1.99	(1.28–3.98)	1.51	(0.94–2.24)		
Not attached	Neither	386 (120)	1.00		1.00				386 (47)	1.00		1.00			
	PI	151 (68)	1.45	(1.07–1.97)	1.33	(0.95–1.86)			151 (36)	2.02	(1.23–3.32)	1.57	(0.92–2.68)		
	Work-oriented intervention (WI) vs. neither														
Comprehensive school	Neither	339 (130)	1.00				1.00		339 (52)	1.00		1.00		1.00	
	WI	162 (71)	1.21	(0.91–1.62)	1.23	(0.92–1.64)	1.16	(0.86–1.55)	162(30)	1.29	(0.78–2.13)	1.30	(0.78–2.15)	1.26	(0.75–2.12)
High school	Neither	187 (88)	1.00				1.00		187 (42)	1.00		1.00		1.00	
	WI	95 (52)	1.27	(0.90–1.80)	1.30	(0.92–1.85)	1.26	(0.87–1.78)	95 (18)	0.81	(0.43–1.52)	0.80	(0.43–1.50)	0.75	(0.40–1.44)
Attached to work	Neither	154 (103)	1.00						154 (48)	1.00		1.00			
	WI	90 (62)	1.08	(0.79–1.50)	1.11	(0.80–1.54)			90 (27)	0.91	(0.51–1.62)	0.94	(0.51–1.72)		
Not attached	Neither	386 (120)	1.00						386 (47)	1.00		1.00			
	WI	173 (64)	1.27	(0.94–1.72)	1.29	(0.95–1.76)			173 (23)	1.14	(0.66–1.95)	1.10	(0.63–1.92)		
	Both interventions vs. neither														
Comprehensive school	Neither	339 (130)	1.00		1.00		1.00		339 (52)	1.00		1.00		1.00	
	Both	60 (27)	1.16	(0.76–1.77)	1.03	(0.67–1.57)	1.03	(0.67–1.58)	60 (18)	2.17	(1.15–4.09)	1.84	(0.95–3.58)	1.90	(0.94–3.85)
High school	Neither	236 (122)	1.00		1.00		1.00		187 (42)	1.00		1.00		1.00	
	Both	49 (34)	1.74	(1.16–2.59)	1.80	(1.17–2.77)	1.66	(1.07–2.57)	49 (17)	1.88	(0.95–3.74)	1.54	(0.74–3.21)	1.41	(0.66–3.05)
Attached	Neither	154 (103)	1.00		1.00				154 (48)	1.00		1.00			
	Both	39 (30)	1.24	(0.82–1.86)	1.20	(0.78–1.85)			39 (24)	3.61	(1.72–7.58)	2.76	(1.26–6.02)		
Not attached	Neither	386 (120)	1.00		1.00				386 (47)	1.00		1.00			
	Both	71 (31)	1.44	(0.97–2.15)	1.51	(0.99–2.29)			71 (11)	1.19	(0.58–2.45)	1.21	(0.57–2.57)		

Model 1: adjusted for age and sex

Model 2: adjusted for age, sex, basic education, diagnosis, and hospital admission

Model 3: adjusted for age, sex, diagnosis, hospital admission, and attachment to work before work disability

The analyses stratified by diagnostic group are presented in Table 4. After complete adjustments, among patients with a psychosis diagnosis, having a plan for a psychotherapeutic intervention (as opposed to those with no plan for either intervention) was associated with entering into employment earlier during follow-up (HR = 2.15, 95 % CI 1.25–3.69). In addition, among patients with a depression diagnosis, having a plan for psychotherapeutic intervention (as opposed to those with no plan for either intervention), was associated with being employed at the end of the 5-year follow-up (HR = 1.84, 95 % CI 1.08–3.15). Among patients in diagnostic classes of bipolar disorder or “other disorder” the intervention plans did not associate with entry to employment or being employed at the end of the follow up.

**Table 4:** Associations between intervention plans and employment outcomes by diagnostic category

Diagnostic category	Planned intervention	Starting employment during follow-up							Being employed at the end of the follow-up						
		n (cases)	Model 1		Model 2		Model 3		n (cases)	Model 1		Model 2		Model 3	
			HR	95 % CI	HR	95 % CI	HR	95 % CI		OR	95 % CI	OR	95 % CI	OR	95 % CI
	Psycho-therapeutic intervention (PI) vs. neither														
Psychosis	Neither	235 (79)	1.00		1.00		1.00		235 (28)	1.00		1.00		1.00	
	PI	31 (19)	2.57	(1.54–4.30)	2.50	(1.47–4.26)	2.15	(1.25–3.69)	31 (3)	0.70	(0.19–2.50)	0.57	(0.15–2.10)	0.45	(0.12–1.72)
Depression	Neither	174 (79	1.00		1.00		1.00		174 (36)	1.00		1.00		1.00	
	PI	151 (89)	1.37	(1.01–1.86)	1.27	(0.92–1.75)	1.34	(0.97–1.86)	151 (55)	2.09	(1.27–3.46)	1.84	(1.09–3.11)	1.84	(1.08–3.15)
Bipolar disorder	Neither	79 (43)	1.00		1.00		1.00		79 (20)	1.00		1.00		1.00	
	PI	38 (25)	1.15	(0.70–1.89)	1.09	(0.66–1.79)	1.16	(0.69–1.93)	38 (10)	0.99	(0.40–2.44)	1.01	(0.40–2.52)	1.05	(0.42–2.66)
Other disorder	Neither	52 (22)	1.00		1.00		1.00		52 (11)	1.00		1.00		1.00	
	PI	30 (15)	1.14	(0.58–2.25)	1.04	(0.51–2.10)	0.57	(0.26–1.25)	30 (8)	1.27	(0.42–3.85)	1.19	(0.37–3.84)	0.89	(0.25–3.17)
	Work-oriented intervention (WI) vs. neither														
Psychosis	Neither	235 (79)	1.00		1.00		1.00		235 (28)	1.00		1.00		1.00	
	WI	113 (50)	1.51	(1.06–2.17)	1.58	(1.09–2.27)	1.40	(0.97–2.03)	113 (17)	1.42	(0.73–2.75)	1.33	(0.67–2.62)	1.18	(0.59–2.37)
Depression	Neither	174 (79)	1.00		1.00		1.00		174 (36)	1.00		1.00		1.00	
	WI	83 (44)	1.20	(0.83–1.74)	1.20	(0.82–1.75)	1.26	(0.87–1.84)	83 (24)	1.56	(0.85–2.86)	1.41	(0.76–2.62)	1.49	(0.78–2.86)
Bipolar disorder	Neither	79 (43)	1.00		1.00		1.00		79 (20)	1.00		1.00		1.00	
	WI	37 (23)	1.46	(0.86–2.48)	1.33	(0.78–2.27)	1.17	(0.67–2.03)	37 (5)	0.51	(0.17–1.52)	0.47	(0.15–1.43)	0.47	(0.15–1.44)
Other disorder	Neither	52 (22)	1.00		1.00		1.00		52 (11)	1.00		1.00		1.00	
	WI	30 (9)	0.71	(0.33–1.57)	0.62	(0.27–1.45)	0.51	(0.21–1.25)	30 (4)	0.51	(0.14–1.86)	0.57	(0.15–2.13)	0.49	(0.11–2.17)
	Both interventions vs. neither														
Psychosis	Neither	235 (79)	1.00		1.00		1.00		235 (28)	1.00		1.00		1.00	
	Both	21 (11)	1.60	(0.85–3.03)	1.64	(0.87–3.11)	1.72	(0.91–3.27)	21 (4)	1.80	(0.60–5.81)	1.81	(0.55–5.98)	1.96	(0.58–6.62)
Depression	Neither	174 (79)	1.00		1.00		1.00		174 (36)	1.00		1.00		1.00	
	Both	51 (32)	1.49	(0.98–2.25)	1.38	(0.90–2.13)	1.28	(0.82–1.99)	51 (19)	2.24	(1.13–4.46)	1.79	(0.87–3.65)	1.66	(0.77–3.58)
Bipolar disorder	Neither	79 (43)	1.00		1.00		1.00		79 (20)	1.00		1.00		1.00	
	Both	13 (6)	0.96	(0.39–2.36)	1.43	(0.75–2.75)	0.66	(0.27–1.64)	13 (5)	2.37	(0.63–8.88)	2.03	(0.52–7.88)	1.95	(0.50–7.71)
Other disorder	Neither	52 (22)	1.00		1.00		1.00		52 (11)	1.00		1.00		1.00	
	Both	25 (12)	1.29	(0.61–2.71)	1.23	(0.57–2.65)	1.49	(0.67–3.30)	25 (7)	1.50	(0.48–4.63)	1.60	(0.50–5.19)	1.60	(0.38–6.68)

Model 1: adjusted for age and sex

Model 2: adjusted for age, sex, basic education, diagnosis, and hospital admissions

Model 3: adjusted for age, sex, diagnosis, hospital admissions, and attachment to work before work disability

## Discussion

We examined whether psychotherapeutic and work-oriented interventions were associated with future employment among young adults who were granted a fixed-term work disability pension due to a mental disorder. Altogether, only one out of five of the young adults was employed at the end of the 5-year follow-up whereas half were on full disability pension. Furthermore, half of them had been employed at some time during the follow-up. The basic idea in granting work disability pension as fixed-term for young adults is to promote return to work or studies after rehabilitation. In our study, the proportion of those with any sort of reported intervention plan was low. Nearly half of young adults had no intervention plan at all.

We found that having a plan for a psychotherapeutic intervention was related to earlier entry into employment during follow-up, as was having a plan for a rehabilitative course or training. The other work-oriented interventions did not contribute to entry into employment. On-the-job rehabilitation was associated with earlier entry into employment after adjustment for sex, age, basic education, diagnosis, and psychiatric hospital attendance, but the association was not significant after also adjusting for attachment to work before work disability, most likely because this type of intervention is in most cases only available for those with previous employment. Furthermore, a plan for both a psychotherapeutic and a work-oriented intervention was associated with being employed at the end of the 5-year follow-up.

Previous research has shown psychotherapy to be effective treatment for mental disorders among young adults [12, 13]. Our findings are in line with this, and also further suggest that psychotherapeutic interventions may enhance competitive employment, most likely through recovery from the disorder, and possibly by reducing fear of returning to work and modifying illness perceptions [25]. The most popular among planned therapies was cognitive-behavioral therapy which was applied in all diagnostic classes, followed by psychodynamic therapy. Among patients with psychotic disorders, also supportive psychotherapy was applied.

Compared to psychotherapeutic interventions, work-oriented interventions are typically shorter and less intensive, and are organized as group interventions as opposed to psychotherapeutic interventions, which most often consisted of individual therapy.

We examined four types of work-oriented interventions and found that when the planned intervention was ‘assessment of work ability and evaluations of rehabilitation needs,’ it did not contribute to future employment. This is understandable since assessment serves better as a part of the larger rehabilitation process [26]. Moreover, social rehabilitation was not associated with employment outcomes. This also is not surprising, since social rehabilitation, e.g. rehabilitative work, is most often planned for those with severe disorder, and the aim is not necessarily to achieve competitive employment.

However, our findings suggest that for young people, rehabilitative courses and training may enhance entry into employment, but that their effect might not be long lasting, as they did not contribute to employment at the end of the follow-up. ‘Vocational rehabilitation’, which most often includes on-the-job rehabilitation such as work trials organized by occupational pension institutes [20], has shown to be effective in Finland. However, this form of rehabilitation is not largely available for young people, due to its requirement of employment history. Internationally, the Individual Placement and Support (IPS) model, which is not common in Finland, has contributed to later finding competitive employment [17–19], also among young adults [20]. Since a large proportion of young adults who are granted fixed-term work disability pension have no job to return to, systematic rehabilitation with close contacts to new workplaces should be organized for these people.

Our result showed that the young adults for whom both a psychotherapeutic and a work-oriented intervention was planned were most likely to be employed at the end of the 5-year follow-up, thus suggesting a long-lasting effect on employment. Previous research also suggests that the best practice for young adults with severe mental illness may be a combination of supportive and demanding elements (like competitive employment) [27]. However, the proportion of young adults who had a plan for both interventions was low, 10 %. Having both interventions in the treatment plan might be a proxy measure for the overall good quality of treatment. Both psychotherapeutic, social and work-related support is needed to counteract the obstacles of successful employment [28], and more collaboration between mental health services and vocational rehabilitation services is needed [29].

The proportion of those employed at the end of the follow-up (22.0 %) was a little lower than that reported in previous study (27 %) of the same age group [21]. These low rates of return to employment may reflect difficult history of illness among young adults with work disability due to a mental disorder [23]. However, it may also indicate poor rehabilitation, and a lack of support and prospects for young people with mental health-related disability to be re-integrated into the labour market in Finland. The fact that 48 % were employed at some point during follow-up, and that only 22.0 % were employed at the end of the follow-up suggests that either permanent employment is difficult to obtain, or the illness hinders possibilities of long-term employment.

The results showing that higher education and attachment to employment prior to work disability predicts later employment suggest that the young adults who benefited most from intervention plans were those with good socioeconomic position.

Among planned therapies cognitive-behavioral orientation was the most popular. The patients with most severe (psychotic) disorder had a plan for psychotherapeutic interventions less often than the patients in the other diagnostic classes. Of the patients with psychotic disorder, only 13 % had a plan for psychotherapeutic intervention. Interventions for patients with psychotic disorder consisted of cognitive or cognitive-behavioral approach, trauma therapy and supportive therapy in which the everyday life of the patient is in focus. Diagnosis-stratified analyses showed that among those with psychosis, a plan for a psychotherapeutic intervention predicted earlier entry into employment, whereas among those with a depression diagnosis, a plan for psychotherapeutic intervention predicted being employed at the end of the follow-up. Psychotic illness may cause difficulties in maintaining employment, and it is also possible that young adults with a psychotic illness who managed to gain employment may have needed more support at the workplace to maintain their employment [30]. Among those with depression, symptoms may hinder employment-seeking in the early phase of the treatment process, but the long-term effects of therapy seem to be beneficial. Psychotherapy funded by national insurance in Finland is more often granted to patients with affective disorders than to those with psychotic disorders [14], who typically have more severe cognitive impairment [31]. Psychotherapy has shown to be more effective for depressed patients than medication alone [32]. The negative beliefs typical in depression, such as fear-avoidance beliefs regarding work [33] and low return-to-work self-efficacy [34] could be optimal targets for psychotherapeutic interventions.

## Strengths and limitations

One important limitation of this study is that it is an observational study: treatment effects are best examined in a randomized controlled trial. However, this study may serve as a basis for future interventions aiming to support young people in their return to the labour market. We were also able to obtain detailed information on planned interventions, and use register-based data on employment statuses through the 5-year follow-up. In addition, we were able to adjust for attachment to employment preceding work disability.

The lack of randomization may have led to selection bias in our study. It is possible that intensive and costly interventions, especially long psychotherapeutic interventions, are offered to individuals who are evaluated as having the potential to benefit from the intervention, while less intensive interventions or no intervention at all are targeted at those with a lower likelihood of finding employment. However, to tackle this problem, we adjusted for diagnostic group, basic education, hospital admission as a proxy measure for severity of illness, and attachment to employment preceding work disability pension, as these factors are known to associate with return to work [6–10]. However, we cannot fully exclude confounding by indication, i.e. the possibility that the patients to whom therapy is planned, are inherently different from the patients without plans.

Another limitation of our study is that we only examined intervention plans, and cannot thus be positive that patients completed their interventions. As planned interventions were documented in treatment and rehabilitation plans which were attached to official work disability pension application, we may expect that the interventions were likely to be started. However, we did not have records on individual patients who may have interrupted their interventions. This may have led to underestimate of the associations of interventions and employment in our study, since the patients who may have interrupted their interventions are dealt as intervention participants.

The differences of work disability benefits across countries limit the external validity of the results. The Finnish system of work disability pension, with rehabilitation compensated by social insurance, differs for example from the system in the United States. Also, even between countries which have disability benefit as a part of social protection systems and spend the highest amount of GDP on disability compensations in EU (e.g. Denmark, Finland, Iceland, Norway, the Netherlands, Sweden, and the UK), there are differences in terminology and eligibility [4].

## Conclusions

Young adults with a long-term psychiatric work disability episode rarely have a recorded plan for rehabilitation in their treatment and rehabilitation plan although psychotherapeutic interventions and a combination of a psychotherapeutic and a work-oriented intervention might be beneficial for promoting employment among them. This observational study serves as a basis for future trials focusing on the integrating young adults with mental disorders into the labour market.

## Abbreviation

ICD-10: International Classification of Diseases tenth revision
